# Association of Body Mass Index and Waist Circumference With Dental Caries and Consequences of Untreated Dental Caries Among 12- to 14-Year-old Boys: A Cross-Sectional Study

**DOI:** 10.1016/j.identj.2021.01.009

**Published:** 2021-02-20

**Authors:** Ravi Kumar Gudipaneni, Rakan Menwer Albilasi, Omer HadiAlrewili, Mohammad Khursheed Alam, Santosh R. Patil, Faisal Saeed

**Affiliations:** a Pediatric Dentistry, Department of Preventive Dentistry, College of Dentistry, Jouf University, Sakaka, Saudi Arabia; b College of Dentistry, Jouf University, Sakaka, Saudi Arabia; c Orthodontics, Department of Preventive Dentistry, College of Dentistry, Jouf University, Sakaka, Saudi Arabia; d Oral Medicine and Radiology, NHDCRI, Bilaspur, India; e Pediatric Dentist and Head of Pediatric Dentistry Department at Specialized Dental Center, MOH, Tabuk, Saudi Arabia

**Keywords:** Dental caries, DMFT index, Obesity, PUFA index, Untreated dental caries

## Abstract

**Objective:**

To evaluate the association between body mass index (BMI) and waist circumference (WC) and dental caries (DC), and the clinical consequences of untreated dental caries (U-DC) among 12- to 14-year-old male public-school adolescents in the Northern Province, Saudi Arabia.

**Methods:**

The demographic and anthropometric measurements of 302 boys 12 to 14 years of age (mean: 12.5 years) were recorded. BMI and central obesity (based on WC) were measured. The decayed-missing-filled teeth (DMFT) index was used to record DC. The pulp involvement, ulceration, fistula, abscess (PUFA) index was used to quantify the clinical consequences of U-DC. Multiple logistic regression analysis was performed to evaluate the risk factors related to DC and clinical consequences of U-DC.

**Results:**

A high prevalence of DC was found in adolescents who were underweight according to BMI and nonobese based on WC (46.7% vs 34.5%). The association between underweight (BMI) and obese (WC) with DC (odds ratio [95% CI]) was 1.91 (0.87, 4.18) and 0.34 (0.18, 0.63), respectively, while with PUFA (adjusted odds ratio [AOR]; 95%CI), it was 1.76 (0.76, 4.09) and 0.19 (0.06, 0.63) respectively. The logistic regression model showed that consuming sugar more than once a day led to a 2.87-fold greater likelihood of DC (AOR [95% CI] = 2.87 [1.68, 4.88]) and a 3.91-fold greater likelihood of mean PUFA score (AOR [95% CI] = 3.91 [2.05, 7.44].

**Conclusion:**

High risks for DC and clinical consequences of U-DC were observed among underweight and nonobese adolescent males. The frequency of sugar consumption was significantly associated with both conditions.

## Introduction

Obesity is a major global epidemic and is associated with increased risk for morbidity and mortality. In the Middle East, 66%-75% of adults and 25%-40% of children are overweight or obese.^1^ Body mass index (BMI) trends of children and adolescents from various high-income nations have remained static from 1975 to 2016, and do not correlating with those seen in adults.^2^ Worldwide prevalence of obesity increased from 0.7% in 1975 to 5.6% in 2016 for girls and from 0.9% in 1975 to 7.8% in 2016 for boys,^2^ while prevalence of moderate and severe underweight children decreased from 9.2% in 1975 to 8.4% in 2016 for girls and from 14.8% in 1975 to 12.4% in 2016 for boys. Prevalence of moderate and severe underweight children was reported in 2017 as highest in India at 22.7% among girls and 30.7% among boys.^2^ Prevalence of obesity was reported to be about 20% or more in Polynesia and Micronesia, the Middle East and North Africa, the Caribbean, and the United States.^2^ In Saudi Arabia, obesity is increasing at an alarming rate due to significant changes in dietary habits and lifestyle practices.^3^

In 2010, national prevalence rates of overweight and obesity in Saudi adolescents were 23.1% and 9.3%, respectively.^4^ Furthermore, dental caries (DC) levels in Saudi Arabia are high per recent reports (2019), when compared globally, and have a prevalence of 64.9% among 12-year-olds and 71.35% among 15-year-olds.^5^

Obesity and DC share common risk factors, including parenting, lifestyle, and psychosocial factors.^6^ Diet plays a significant role for both and includes poor eating habits such as increased frequency and excessive consumption of high-calorie fermentable carbohydrates and cariogenic junk food.^7^^,^^8^ Other factors are age and sex.^9^ It has been reported that dietary factors, oral hygiene practices, and socioeconomic status (SES) are more significant risk factors for DC than for obesity.^10^^,^^11^

Previous studies focused on the associations among DC, BMI, and nutritional status have reported conflicting findings. Although some did not find evidence of associations,^12^ others have shown that obesity increases the probability of DC.^13^ Furthermore, a significant relationship was found between being underweight and DC.^14^^,^^15^ Undernutrition and overweight/obesity are each categories of malnutrition.^16^ The coexistence of malnutrition with overweight and obesity is a global challenge for individuals, families, communities, and countries.^16^ Moreover, a significant association between low BMI and the consequences of untreated dental caries (U-DC) in children has been reported.^17^

In Saudi Arabia, epidemiological studies have been conducted in different regions to explore the link between obesity and DC; these have reported conflicting results. A positive association was found in Al-Kharj city,^18^ Jeddah,^19^ the Riyadh region,^20^ and Taif.^21^ In contrast, an inverse association was observed in the Qassim region,^22^ the Medina region,^23^ and Jazan city.^24^ It has been observed that DC prevalence^5^ and obesity status^3^are high in Saudi Arabia when compared to global values. However, there is a lack of agreement about the association between nutritional status and DC in reported studies.

Severely decayed teeth have significant impact on the overall health, nutrition, height, and weight of children and lead to discomfort, pain, sleep problems, learning disabilities, and absence from school.^13^ Because of the common risk factors discussed, it is essential to verify the associations between nutritional status and DC and the clinical consequences of U-DC, along with associated risk factors. There have been few reports from the northern region of Saudi Arabia that have evaluated this relationship. Hence, our study investigated associations among BMI, central obesity (assessed by weight circumference [WC]), and DC, including the clinical consequences of U-DC. We also explored the impacts of SES, parent education levels, frequency of tooth brushing, and frequency of sugar intake on DC and the clinical consequences of U-DC, among 12- to 14-year-old male public-school adolescents.

## Methods

This descriptive, cross-sectional study was conducted between December 2018 and March 2019 in Aljouf province, in the northern region of Saudi Arabia. Ethical approval was obtained from the Local Committee of Bioethics, Jouf University, Saudi Arabia (LCBE Approval No: 7-20-3/40). Written informed consent was obtained from the parents or caregivers of participants after they were provided an explanation of the study objectives. All procedures were in full accord with the Declaration of Helsinki. The study enrolled 12- to 14-year-old male public high school adolescents, who also provided written informed consent. Those suffering from medical illness or who failed to provide consent, as well as female adolescents, were excluded from the study.

The Aljouf region has 3 governorates: Sakaka, Qurayyat, and Dumat Al-Jandal. Multistage random sampling was used to recruit the participants. In the first stage, 3 schools from each district were chosen randomly from a list of schools in the region. In the second stage, systematic random sampling was used to select adolescents from the schools’ admission lists.

To calculate sample size, the prevalence of overweight and obesity in the population was considered to be 23% based on previous population-based studies.^25^ The size of the sample was then calculated using OpenEpi software,^26^ with a precision of 5%, type 1 error of 5%, and a 95% CI. The study sample size needed was initially determined to be 272 participants. After estimating an expected dropout rate of 10%, the final minimum sample size required was determined to be 302 participants.

### Data collection

All participants received a semistructured questionnaire to be filled out by parents or caregivers. The self-administered questionnaire was used to obtain sociodemographic data, including age, tooth brushing frequency, parent education level, SES, and frequency of sugar consumption. SES was defined as low (monthly income of ≤3,000 Saudi Riyals [SAR]), middle (monthly income of >3,000 but <10,000 SAR), and high (monthly income of ≥10,000 SAR).^27^ The reliability of the questionnaire (α = 0.8) was calculated by requesting 30 randomly selected parents to complete it again during a face-to-face interview after 2 weeks.

### Anthropometric examination

The height of each participant was measured, without shoes, to the nearest 0.5 cm, using a stadiometer. The body weight of participants was measured while they wore minimal clothing, to the nearest 0.1 kg, on a digital scale. The BMI (kg/m^2^) of each child was calculated from body weight and height. The results were plotted on BMI percentile curves provided by the US Centers for Disease Control and Prevention.^26^ Based on their BMI, children were classified into 4 categories: underweight (BMI for age <5th percentile), normal weight (BMI for age between the 5th and 84th percentile), overweight (BMI for age between the 85th and 95th percentile), and obese (BMI for age >95th percentile). Central obesity was assessed by measuring WC to the nearest 0.5 cm while the participant was in a standing position and using a nonelastic measuring tape. The reference point chosen was the highest point on the iliac crest at the end of a gentle expiration. Participants were classified as obese when WC was >90th percentile for age and as nonobese when WC was <90th percentile for age.^28^

### Caries examination

Oral examinations of the participants were conducted at their schools. Individual students were seated on a chair, and the examination was carried out using plane mouth mirrors under artificial light, World Health Organization (WHO) probes, disposable gloves, masks, and wooden tongue depressors.

No radiographs were taken to diagnose DC lesions. DC status was determined using the decayed-missing-filled teeth (DMFT) index,^29^ which is most often used to measure DC experience. The DMFT index cannot ascertain clinical consequences of U-DC (ie, involvement of the pulp, abscess, and sinus/fistula). Because U-DC affect the growth, development, and quality of life of children and adolescents,^30^ we used the pulp involvement, ulceration, fistula, and abscess (PUFA) index^17^ to establish these.^31^ The PUFA index also allows quantification of the clinical consequences of U-DC for epidemiological studies. It was calculated in the same cumulative manner as the DMFT index.^31^

A single investigator was involved in both the dental examinations and anthropometric measurements. Intraexaminer validity was calculated by re-examining 30 participants in 1 randomly selected school; the results showed κ values of 0.90 for the DMFT index and 0.85 for the PUFA index. Measurements for waist, height, and weight were recorded twice for each child, and the average was used for analyses.

### Statistical analysis

The data collected were analysed using χ^2^ tests and multiple logistic regression according to the Statistical Package for the Social Sciences version 24.0 (IBM Corp.). Multiple logistic regression was performed to identify factors associated with BMI, central obesity, and DC, including the consequences of U-DC. First, a simple logistic regression was conducted to screen for significant variables (*P* < .25). Subsequently, multiple logistic regression was done to adjust for the effects of other variables. Multiple logistic regression analysis was performed using 3 automatic methods (forward logistic regression, backward logistic regression, and forward conditional logistic regression). The method with the highest number of significant variables (*P* < .05) was chosen, after which the enter method was used to obtain the final model. Model fitness was assessed using the Hosmer–Lemeshow test.

## Results

The total enrolment was 302 participants. Mean age was 12.57 ± 0.50 years. The participants were almost equally distributed throughout the province's regions: Sakaka (32.4%), Qurayyat (32.7%), and Dumat Al-Jandal (34.7%). Participants came from 9 separate schools, randomly selected (3 schools per governorate), to form as representative a sample of the Aljouf province as possible.

The overall prevalence of the presence of 1 or more decayed tooth (D ≥ 1) and 1 or more pulpally involved tooth (P ≥ 1) among the study population was 28% and 26%, respectively. The distribution of the study sample according to SES, level of parent education, frequency of tooth brushing, frequency of sugar intake, BMI, and the central obesity associated with DC and the clinical consequences of U-DC is shown in Table 1. We observed that DC were highly prevalent among those of high SES (45.5%), those consuming sugar more than once per day (38.9%), those with a low parental education level (31.5%), those who were underweight according to BMI (46.7%), and those who were nonobese by WC (34.5%). The overall mean of the consequences of U-DC as assessed by the PUFA score was 0.38 (0.78), and the highest mean PUFA scores were found in the underweight adolescents (0.60 [0.81]).

**Table 1:** Dental caries status and PUFA score according to study variables.

Characteristics	*N* (%)	Caries status			PUFA Score Mean (SD)
		Presence (D ≥ 1) *N* (%)		Absence (D = 0) *N* (%)	
Socioeconomic status					
Low	156 (51.7)	46 (29.5)		110 (70.5)	0.44 (0.74)
Middle	135(44.7)	34 (25.2)		101 (74.8)	0.30 (0.84)
High	11(3.6)	5 (45.5)		6 (54.5)	0.45 (0.52)
**Frequency of sugar intake**					
Once per day	171(56.6)	34 (19.9)		137 (80.1)	0.27 (0.58)
More than once per day	131(43.4)	51 (38.9)		80 (61.1)	0.52 (0.96)
**Frequency of brushing**					
Once a day	23(7.6)	8 (34.8)		15 (65.2)	0.70 (1.72)
Twice a day	37(12.3)	12 (32.4)		25 (67.6)	0.24 (0.43)
Irregular	242(80.1)	65 (26.9)		177 (73.1)	0.37 (0.67)
**Parental education**					
Primary	143(47.4)	45 (31.5)		98 (68.5)	0.47 (0.76)
Secondary	62(20.5)	13 (21.0)		49 (79.0)	0.18 (0.39)
Higher	87(30.3)	27 (27.8)		70 (72.8)	0.37 (0.96)
**BMI groups**					
Underweight (less than 5th percentile)	30(9.9)	14 (46.7)		16 (53.3)	0.60 (0.81)
Normal weight (5th to 84th percentile)	178(58.9)	56 (31.5)		122 (68.5)	0.39 (0.68)
Overweight (85th to 95th percentile)	54(17.9)	7 (13.0)		47 (87.0)	0.04 (0.19)
Obese (greater than 95th percentile)	40(13.2)	8 (20.0)		32 (80.0)	0.60 (1.34)
**Central obesity measured by WC**					
Nonobese (less than the 90th percentile)	203(67.2)	70 (34.5)	133 (65.5)		0.43 (0.71)
Obese (more than 90th percentile)	99(32.8)	15 (15.2)	84 (84.8)		0.26 (0.90)
Total	302(100)	85 (28.1)	217 (71.9)		0.38 (0.78)

BMI = body mass index; PUFA = pulp involvement, ulceration, fistula, and abscess; SD = standard deviation; WC = waist circumference

Factors associated with DC were analysed using logistic regression as shown in Table 2. Among the covariates, frequency of sugar consumption, BMI, and central obesity were found to be statistically significant (*P* value < .25). Participants who consumed sugar more than once per day were 2.57 times more likely to have DC than those who consumed sugar only once per day (crude odds ratio [COR] = 2.57; 95% CI 1.54-4.30). Those who were underweight by BMI were 1.9 times more likely to have DC than those with normal weight BMI (COR = 1.91; 95% CI0.87-4.18). Those who were overweight by BMI were 68% less likely to have DC than those who were normal weight by BMI (COR = 0.32; 95% CI 0.14-0.76). Participants who were obese were 45% less likely to have DC than those who had normal weight (COR = 0.55, 95% CI 0.24-1.26). In the case of central obesity, obese individuals were 66% less likely to have DC than those who were nonobese (COR = 0.34; 95% CI 0.18-0.63).

**Table 2:** Factors associated with dental caries among adolescents.

Variable	COR (95% CI)	*P* value	AOR (95% CI)	*P* value
**Socioeconomic status**				
Low	1			
Medium	0.81 (0.48, 1.35)	.413		
High	2.0 (0.58, 6.86)	.274		
**Frequency of brushing**				
Once	1			
Twice	0.90 (0.30, 2.70)	.851		
Irregular	0.69 (0.28, 1.70)	.418		
**Frequency of sugar intake**				
Once	1		1	
More than once	2.57 (1.54, 4.30)	<.001	2.87 (1.68, 4.88)	<.001
**Parent education**				
Primary	1.19 (0.68, 2.10)	.547		
Secondary	0.69 (0.32, 1.46)	.332		
Higher	1			
**BMI**				
Normal weight	1			
Underweight	1.91 (0.87, 4.18)	.107		
Overweight	0.32 (0.14, 0.76)	.010		
Obese	0.55 (0.24, 1.26)	.155		
**Central obesity**				
Obese	0.34 (0.18, 0.63)	.001	0.30 (0.16, 0.57)	<.001
Nonobese	1		1	

AOR = adjusted odds ratio; BMI = body mass index; CI = confidence interval; COR = crude odds ratio.

In multiple logistic regression, only the frequency of sugar intake and central obesity remained statistically significant predictors of DC. Those who consumed sugar more than once per day were 2.87 times more likely to develop DC (adjusted odds ratio [AOR] = 2.87;95% CI 1.68-4.88). After adjustment, obese adolescents, based on WC, were 70% less likely to develop DC (AOR = 0.30; 95% CI 0.16-0.57). Model fitness was assessed using the Hosmer–Lemeshow test (*P* value = 1.00), with an overall classification of 71.9%, and the area under the curve (AUC) was 67.6%. Hence, the final model was considered to adequately fit the data.

Factors associated with the clinical consequences of U-DC are shown in Table 3. Among these, SES, frequency of sugar consumption, central obesity, parental education level, and BMI were considered statistically significant after simple logistic regression (*P* value < .25). Hence, these factors were included in the multiple logistic regression to adjust for the effects of other variables. Participants who consumed sugar more than once per day were 2.11 times more likely to have clinical consequences of U-DC than those who consumed sugar only once per day (COR = 2.11; 95% CI 1.25-3.55). In multiple logistic regression analysis, those who consumed sugar more than once per day were 3.9 times more likely to have clinical consequences of U-DC (AOR = 3.91; 95% CI 2.05-7.44). In terms of parental education level, those with parents at a primary school level of education were 11% more likely to have clinical consequences of U-DC than those who had parents with a higher level of education (AOR = 1.11; 95% CI 0.56-2.22). In terms of BMI, the underweight group were 1.76 times more likely to have clinical consequences of U-DC than the normal weight group (AOR = 1.76; 95% CI 0.76-4.09). For central obesity, those who were obese were 81% less likely to have clinical consequences of U-DC than those who were nonobese (AOR = 0.16; 95% CI 0.06-0.63). The fitness of the model was assessed using the Hosmer–Lemeshow test (*P* < .001), with an overall classification of 72.5%, and the area under the curve was 78.0%. Hence, the final model was considered to fit the data adequately. However, only the Hosmer–Lemeshow *P* value was less than .05 and, therefore, statistically significant; the other fit indices exceeded the cutoff value

**Table 3:** Factors associated with clinical consequences of untreated dental caries (PUFA) among adolescents.

Variable	COR (95% CI)	*P* value	AOR (95% CI)	*P* value
**Socioeconomic status**				
Low	1			
Medium	0.50 (0.29, 0.86)	.013		
High	1.82 (0.53, 6.25)	.342		
**Frequency of brushing**				
Once	1			
Twice	1.53 (0.41, 5.68)	.528		
Irregular	1.78 (0.58, 5.43)	.310		
**Frequency of sugar intake**				
Once	1		1	
More than once	2.11 (1.25, 3.55)	.005	3.91 (2.05, 7.44)	<.001
**Parent education**				
Primary	1.77 (0.98, 3.22)	.060	1.11 (0.56, 2.22)	.759
Secondary	0.78 (0.35, 1.76)	.549	0.26 (0.10, 0.70)	.007
Higher	1		1	
**BMI**				
Normal weight	1		1	
Underweight	1.57 (0.71, 3.49)	.266	1.76 (0.76, 4.09)	.189
Overweight	0.09 (0.02, 0.39)	.001	0.21 (0.04, 1.05)	.058
Obese	1.01 (0.48, 2.14)	.978	0.73 (0.03, 0.50)	.045
**Central obesity**				
Obese	0.35 (0.19, 0.66)	.001	0.19 (0.06, 0.63)	.006
Nonobese	1		1	

AOR = adjusted odds ratio; BMI = body mass index; CI = confidence interval; COR = crude odds ratio; PUFA = pulp involvement, ulceration, fistula, and abscess.

## Discussion

In our study, high rates of DC and clinical consequences of U-DC were found in adolescents who were underweight according to BMI, as well as in those who were nonobese according to WC. The consumption of sugar more than once per day was significantly associated with both conditions, after controlling for potential effects from other covariates.

Studies in different regions of Saudi Arabia have reported conflicting results regarding the association between obesity and DC. These could be due to differences in methodology or sample characteristics. However, many used only BMI when defining obesity.^32^ Even though BMI is most commonly used to assess obesity, it does not differentiate between body fat and lean body mass.^33^ In our study, both BMI and WC were measured to overcome this limitation. WC is considered an accurate anthropometric indicator of central adiposity in children and more sensitive and specific in assessing obesity than BMI and waist-to-hip ratio.^34^ Therefore, our data provide baseline references for future studies investigating associations between nutritional status and risk factors for DC and the consequences of U-DC in this population.

One recent report^16^ stated that malnutrition is related to DC and underweight children are more likely to have a greater number of decayed teeth and related clinical consequences than normal-weight children. They found that a high frequency of sugary food consumption alters the effects of nutritional status on U-DC. Another found that chronic U-DC had a negative effect on BMI in children, and this effect was higher in younger children than in the older age group.^35^ Moreover, DC and U-DC clinical consequences have been found to be important predictors of poor school performance in low-income adolescents.^36^ There is a variable relationship between DC and nutritional status, which is mainly determined by family sociodemographic characteristics.^37^ Developed countries have an unequal distribution of DC than developing countries, which could modify the risk factors associated with DC.^38^

In this study, children of parents with a low level of education had 1.19 times higher risk of DC than those of parents with a high educational level. Other studies have reported similar findings.^39^ Moreover, the association between low educational background and having a DMFT score > 0 was found significant in developed countries.^38^ This has been explained by the fact that parental education level determines family income and influences access to home and professional preventive care measures, such as fluoride toothpaste, dental floss, low-caloric sugar-free diets, and privately paid dental sealants.^40^ In addition, parental education background affects factors such as oral health literacy and behaviour, dietary and oral hygiene practices, and frequency and pattern of dental health services utilisation.^38^

In the present study, the underweight group was 1.9 times more likely to have DC than normal-weight or obese groups, and 1.7 times more likely to have clinical consequences of U-DC than the normal-weight group. However, other studies^41^^,^^42^ have demonstrated low DC levels in obese children, as well as a high prevalence of consequences of U-DC among underweight children and children with delayed growth. Malnutrition has been associated with DC among Brazilian children from low-income families.^16^ It has been posited that the association between underweight status and DC in children could affect enamel maturation and composition, as well as tooth morphology and reduced salivary flow and buffering capacity, which, in turn, increase susceptibility to DC.^43^ In contrast, the low prevalence of DC among obese children has been explained by increased stimulation of salivation due to increased food consumption, which buffers pH lowering and acts as a mechanical cleanser.^44^

It is important to note that pain and oral infection resulting from U-DC have direct and indirect effects on the nutritional status of a child, leading to underweight, malnutrition, and growth failure.^15^^,^^45^ Poster et al^43^ reported that salivary gland hypofunction and changes in salivary composition, as well as enamel hypoplasia, were associated with both DC of the primary teeth and early childhood malnutrition. The high impact of the clinical consequences of U-DC has been previously reported in socioeconomically deprived and underweight children.^46^ Furthermore, a significant association between the clinical consequences of U-DC and low BMI was reported among Filipino children.^17^ A high prevalence of the clinical consequences of U-DC in primary teeth was associated with underweight status in Saudi preschool children,^15^ and it was determined that the pain and infections associated with U-DC could have affected the growth and development of these children.^15^ The association between low weight and U-DC is considered to be an important determinant of childhood developmental disabilities, although this continues to be an often neglected factor.^14^ Pulpal involvement was more commonly reported as a clinical consequence of U-DC among our study population than other components of the PUFA index. It was assumed that the attitude and behaviour of adolescents and their parents towards utilising dental services in the northern region of Saudi Arabia could be one explanation. Access to oral health services and the use of fluoridated substances were considered to be potential effect modifiers.^47^ Other regions of Saudi Arabia have reported that poor oral hygiene practices, lack of parental guidance, and appropriate dental health knowledge, together with frequent exposure to cariogenic foods, in addition to sociodemographics, were the main risk factors for U-DC.^48^

In terms of central obesity measured by WC, the nonobese group was 2.9 times more likely to have DC and the clinical consequences of U-DC than the obese group. However, when included in the multiple logistic regression analysis, the underweight group did not reach statistical significance.. Given that there was no significant interaction between BMI status and WC for either DC or the consequences of U-DC, the underweight variable may have lost statistical power, considering that it evaluated the same characteristic as WC.

In our study, a slightly higher frequency of DC was found among obese adolescents when obesity status was measured by WC than by BMI. This indicates that the association between BMI and DC can underestimate the prevalence of DC. Moreover, in the present study, it was observed that a higher number of adolescents were classified as obese when obesity was measured by WC than when measured by BMI. Further studies are needed to support our results; it would be valuable to determine whether BMI or central obesity is the optimal risk indicator for DC in epidemiological studies and preventive programs.

The present cross-sectional study had limitations As this was an observational study, causal relationships could not be determined. Our study involved only males and had a minimum sample size because of accessibility limitations in the female population. Other limitations, such as the quality of sugar and specific lifestyle habits, have not been considered and could act as confounders. Furthermore, underweight adolescents comprised only 9.9% of the study sample, which might have compromised the representativeness of these participants. In the future, prospective longitudinal studies with a larger sample size should be performed to confirm our findings. Additionally, further studies will be essential to determine whether these results are the same in female adolescents.

## Conclusion

Our study provides an understanding of the relationship between BMI and DC, including the clinical consequences of U-DC in adolescents in a northern province of Saudi Arabia. Those who were underweight by BMI and nonobese by WC had a high prevalence of DC and clinical consequences of U-DC. Frequency of sugar consumption was mainly associated with increased prevalence of DC and their clinical consequences when left untreated. There is a need for oral health prevention strategies, such as behavioural and dietary modifications, to prevent DC and the clinical consequences of U-DC. Oral health care providers and policy makers should target intervention programs accordingly.

## Funding

This research did not receive any specific grant from funding agencies in the public, commercial, or not-for-profit sectors.

## Conflict of interest

None disclosed.
